# Improving the Understanding of Psychological Factors Contributing to Horse-Related Accident and Injury: Context, Loss of Focus, Cognitive Errors and Rigidity

**DOI:** 10.3390/ani6020012

**Published:** 2016-02-15

**Authors:** Jodi DeAraugo, Suzanne McLaren, Phil McManus, Paul D. McGreevy

**Affiliations:** 1 Faculty of Health, Department of Psychology, Federation University, University Drive, Mount Helen, Victoria 3350, Australia; s.mclaren@federation.edu.au; 2 School of Geosciences, University of Sydney, Room 435, F09, Madsen Building, New South Wales 2006, Australia; phil.mcmanus@sydney.edu.au; 3 Department of Veterinary Science, University of Sydney, Room 206, R.M.C. Gunn Building, New South Wales 2006, Australia; paul.mcgreevy@sydney.edu.au

**Keywords:** human–horse risk, context-specificity, attention, cognitive error, self-reference

## Abstract

**Simple Summary:**

There is a high risk of injury for people involved with horses in their work or recreational pursuits. High risks are particularly evident for racing employees and veterinarians. Elevated risks of injury may be associated with misjudging how to handle situations, reduced attention caused by distractions, taking a general view, and failing to consider other strategies that may reduce risks. To improve safety for humans and horses, it is important to identify safety strategies that are flexible, focused and specific.

**Abstract:**

While the role of the horse in riding hazards is well recognised, little attention has been paid to the role of specific theoretical psychological processes of humans in contributing to and mitigating risk. The injury, mortality or compensation claim rates for participants in the horse-racing industry, veterinary medicine and equestrian disciplines provide compelling evidence for improving risk mitigation models. There is a paucity of theoretical principles regarding the risk of injury and mortality associated with human–horse interactions. In this paper we introduce and apply the four psychological principles of context, loss of focus, global cognitive style and the application of self as the frame of reference as a potential approach for assessing and managing human–horse risks. When these principles produce errors that are combined with a rigid self-referenced point, it becomes clear how rapidly risk emerges and how other people and animals may repeatedly become at risk over time. Here, with a focus on the thoroughbred racing industry, veterinary practice and equestrian disciplines, we review the merits of contextually applied strategies, an evolving reappraisal of risk, flexibility, and focused specifics of situations that may serve to modify human behaviour and mitigate risk.

## 1. Introduction

Safety risks for people working with, riding, or otherwise being in proximity to horses are well documented. These include injury or death and, across the different equestrian disciplines, horse riding is widely considered a high risk activity [1,2,3,4,5,6,7,8,9,10,11,12,13,14,15], as is working with larger animals [16,17,18]. The literature identifies racing industry employees, veterinarians and equestrian disciplines at particular risk of injury or death [1,2,3,4,5,6,7,8,9,10,11,12,13,14,15,16,17].

At especially high risk are thoroughbred racing industry employees [1,2,6,7]. For example, in Australia, work-related injury claims from 2002 to 2010 due to injuries sustained from working with horses averaged $A9 million per year for workers in the racing industry [7]. There are high risk injuries [1,2,6,7] in the racing industry with high insurance costs [6,7]. Curry *et al.* [7] reported that 39% of race-day incidents accounted for 52% of the insurance costs. Race-day insurance costs were on average higher ($A33,756) than non-race-day incidents (which averaged $A20,338). Curry *et al.* study reported that 49% of the sample had injuries to lower and upper limbs, with fractures also prevalent [7]. However, head injuries were less common (5.3% of fall injuries and 2.7% of no-fall injuries) but related to greater monetary costs and more days absent from work [7]. Forero Rueda *et al.* [1] reported rates of concussion and head injuries in flat and jumps jockeys in Ireland (23 per 1000 falls), France (32 per 1000 falls) and Britain (19 per 1000 falls) that seemed lower than those reported by Curry *et al.* [7]. Injuries per fall rates from flat racing were between 33% and 44% [1]. Jumps racing per ride had the highest injury rates for all jockeys (*i.e.*, both amateurs and professionals) [1,2] and, in Australia, 17.4%–21.9% of compensation claims were jumps racing jockeys [7]. The average monetary claim for jumps racing jockeys was $45,831 compared to $24,672 for flat racing jockeys [7]. An Australian study by Cowley *et al.* [6] reported that track riders and stable attendants accounted for most of the worker compensation claims (71%) and, among them, 72% cited horse-related injuries due to falls from horses. Other injuries reported were being kicked, struck, hit, crushed or pushed by horses. Fractures and contusions were listed as the primary injuries [6].

Elevated risks of injury are related to the type of race, distance of the race, experience of the horse and jockey (*i.e.*, apprentice and amateur jockeys) [1,2,7]. Hitchens *et al.* [2] considered jockey, horse and environmental variables as influences on the risks in racing. Thus, the racing data show variation in the risk profile of injury, but there are suggestions that inexperienced jockeys, track riders and less accomplished horses elevate the risks of injuries.

Other horse-related professionals at risk appear to be veterinarians. Data indicate that most large-animal vets were at an increased risk of significant injuries compared to companion animal vets [17]: 51% receiving injuries in the workplace that affected the span of their professional working life, and 26% having sustained injuries in the preceding year. Lucas *et al.* [11] cited evidence that the most common injuries (79%) reported by vets were sustained either by being kicked or struck by horses. This was similar to injuries cited for veterinary and animal science students (*n* = 260): foot/ankle (39.1%), upper leg/knee (34.8%), and hand (13%) being the most common areas injured [12]. The most prevalent ways students were injured were by being trampled or kicked by a hind limb (30.4%), bitten (13%), or falling when riding (8.7%). The most common nature of the 31 injuries reported were bruising/soft tissue injury (91.3%), open wound (17.4%), muscle or tendon injury (8.7%).

Similarly, equestrian disciplines have high rates of injury [3,4,5]. The equestrian risk (11.2%) was almost equivalent to injuries from all-terrain vehicle injuries (12.2%) and 1.4 times greater than sports-related injuries [5]. Mayberry *et al.* [4] reported that injury is common and serious, most notably in the first one hundred hours of gaining experience. The risk of serious injury for equestrians was reported as 1 in 5 [4]. Despite professionals reporting lower rates than novice riders due to their higher skill level, they were at great risk (94%) of sustaining an injury during their career [4]. Lim reported that less experienced or younger riders were more likely to be hospitalised than experienced riders, possibly because older riders rolled to break the fall [3]. A larger scale review by Hawson *et al.* [10] of the human–horse injury literature stated that the most common risks to non-veterinarians were from falling or being thrown from a horse. Head injuries including concussion and brain injuries are most likely when helmet use is limited [3,6]. Therefore, despite the overall high risk of injury to equestrians, from the limited evidence for injuries requiring hospitalisation it appears that less experienced novice and non-helmeted riders are at greater risk. However, even career professionals are exposed to significant risk with a rate of at least one injury in their career, so exposure to horses seems to be implicated also. These data provide compelling evidence of the need for clear determinants of risk-mitigation models that can better address the risky nature of working with horses and other large animals.

Thompson *et al.* [13] summarised many of the most prevalent horse-related risks for people as including the inherent risks themselves, characteristics of the horse, characteristics of the rider, and influential factors evident in the broader horse culture. Some of these risks can be reduced by the design of thoroughbred facilities such as stud farms, auction venues and racetracks [19], but the horse and rider risk variables remain [2,3,4,5]. An appreciation of these risks should prompt researchers across various disciplines to the study of horse-related risks for people, including track or equestrian-centre employees, handlers and riders. To address the dearth of psychological equestrian risk literature, it is salient to include already established and evidence-based psychological variables that could potentially mitigate risk of injuries to humans during their interactions with horses.

In the current paper, we specifically use some of Michael Yapko’s [20,21,22] evidence-based ideas of cognitive processes and his emphasis on the effects of one’s quality of focus on eventual outcomes as a theoretical anchor. Yapko is best known for combining focused states of awareness (*i.e.*, paying attention under hypnosis) with cognitive therapy to alleviate depression, anxiety and other negative emotional states to assist people in making associations with existing resources or developing new skills [20,21,22]. He includes attributional style as an influence on the development of cognitive therapy and hypnosis [23]. He has also used focused awareness, such as hypnosis and cognitive therapy, to recalibrate dangerous health behaviours, which essentially recalibrates the cognitive, emotional, physical and behavioural risks for destructive human behaviours [22]. Focus is central to his work because it amplifies awareness that is an integral part of change and this enhances processing new information [20,21,22]. In essence, he focuses on changing psychological process errors (*i.e.*, association rather the dissociation and being specific as opposed to being global in some circumstances) rather than the content (*i.e.*, basis of the person’s story). It is evidence-based and merits consideration in the current context of reducing risks in human–horse interactions, which is a novel arena for the application and extension of his work. There is useful literature on naturalistic decision-making [24,25], in situations that require rapid responses, but this lies beyond the scope of the current article. Indeed, it makes sense to apply established psychological theoretical principles with effective clinical outcomes supported by a body of evidence, to a novel target such as human–horse risks.

Yapko suggests psychological targets, such as context (*i.e.*, situational factors shaping responses), focus (*i.e.*, the direction and quality of one’s focus) and cognitive style (*i.e.*, how one assimilates and integrates information), as processes for understanding the mitigation or elevation of the risk for humans who engage in behaviours that may be detrimental to their health. This cognitive and contextual information can be applied to the area of risks arising for employees working in the horse racing industry (e.g., jockeys, track riders, attendants and veterinarians) and others who have contact with horses across the equestrian disciplines.

In the following sections of this paper, we consider whether context, loss of focus, global cognitive style and the application of self as the frame of reference are important and how they can relate to each other, in exacerbating and managing risks.

## 2. The Relevance of Context in the Changing Profile of Risk

The importance of contextual relevance is evident when some purposeful strategy may work well in one particular area or interaction, yet the same strategy may fail when applied in a second situation when the context does not support its use [20,22]. The effective application of a given strategy would need to account for specific cues perceived by the rider, from the particular context of the immediate human–horse interactions. A strategy from a previous context may or may not work in the current one.

Assessing the contextual cues from humans and horses associated with risky behaviour and then selecting the appropriate (*i.e.*, safe, effective) strategy is paramount. It is possible that, when working with horses, making decisions within a short time-frame may also be a paramount consideration (e.g., quick and efficient decision-making skills) that could help riders respond more effectively and thereby prevent accident or injury. This point is supported by Hausberger *et al.* [26], who indicated that people require different skills for different contexts. They separated the skills necessary for handling horses well from the skills of riding horses well. As such, racehorse attendants’ duties, which include animal care, grooming, preparation of the horses, cleaning stables and horse-handling, differ substantially from track riders’ and jockeys’ occupational requirements [6]. Furthermore, veterinarians’ skills are vastly different to the skills of others in the horse industry. Veterinarians assess and treat equine medical problems, such as attending to horses in crushes, examining horses that may be in pain, distressed or in unusual circumstances (e.g., traffic or accidents during transportation in a float), all of which pose different risks to personnel [27]. In summary, different jobs require different skills that include job-specific assessments of human–horse interactions. Arguably, risk mitigation can be progressive, and ranking the skill-sets for different duties relies on re-evaluation of risk across different circumstances, situations and with different horses.

Changes in context are of great relevance in risk management at the human–horse interface. They could simply represent a shift from the horse’s home environment to an unknown or less familiar environment (e.g., racecourse, veterinarian facility or competition venue). Cowley *et al.* [6] state that, in a shared track-riding environment, occupational hazards can be especially prevalent because trainers have less influence over the environment than they do in a private context. Equine responses reflect the familiarity, but also predictability, of their current environment. As a prey species, horses are flight animals that are characterised by unpredictability or their instinctive need for safety [13,28]. Three examples are provided in Table 1.

**Table 1:** Examples from track-work riders and jockeys, equine veterinarians and equestrian competitors that illustrate the relevance of context and purposeful effective strategies that can increase the risk of injuries.

Track-Work Riders and Jockeys	Equine Veterinarians	Equestrian Competitors
Examples of how awareness of the context can be used to recalibrate risk		
A horse in a racecourse environment may be adrenalinised on race day by the atmosphere at the track. The crowd, noise, speakers, barriers, and other horses could prompt a flight response in the horse, particularly with a younger horse just introduced to the new environment. Horses are often kept moving (*i.e.*, walking) to cope with the stressful atmosphere. An unpredictable response from a person in the crowd, such as a flag flying near the horse, could prompt a startle response and flight reaction. The racing attendant may use the focus of the horse to divert its attention from the flag, move the horse away, or habituation to flags could be undertaken prior to race day to prepare the horse.	A horse may not have been exposed to a crush prior to attending a vet‘s premises. It may be in pain and require treatment. The horse may trial running backwards or sideways, and either kick out or barge over the handler if it fears being put in the crush. Some vets may, with the owner‘s consent, sedate the horse for safety of the horse and personnel. A contextual alternative could be to use clicker training (*i.e.*, positive reinforcement with food) or exploring the environment with wither scratching, if it is in the training repertoire of established responses. A poorly chosen strategy, such as the handler using a whip for punishment, could produce disastrous, noxious and fear-related results and potentially exacerbate the flight response contributing to possible injuries to those involved.	When a competition horse in the home environment sees an unfamiliar object, such as a camera on a tripod, it may seek to avoid the object and need reassurance, such as calm verbal responses and wither scratching and/or (if under saddle) leg cues to move past the object [27]. The same horse in a competition environment may require time and free exploration to habituate to novel objects. It may also need reassurance before approaching such objects [27]. Thus, the strategy chosen by the rider could vary according to the environmental conditions and potential reactivity or flight response of the horse. Forcing horses past novel objects in the competition atmosphere may exceed their tolerance threshold, increasing the risk of a flight response, and potential for human/animal injury.

## 3. The Counter-Balance of Focus or Loss of Focus

The second variable to consider as part of a risk-mitigation model relates to loss of human focus. The definition of dissociation is the ability to separate a broad (*i.e.*, global) experience into its component parts and reduce awareness [22]. Essentially, dissociation is a reduction in the direction and quality of focused attention. Yapko [22] emphasises that dissociation is a neutral term. That said, he makes the point that dissociation can be used as a negative or positive process and that it ultimately depends upon the context and specificity of its application [22]. A person’s attention may be internally oriented (*i.e.*, they may be thinking about the other tasks, past events or daydreaming), or they may be distracted and diverting their attention to another task in their environment. It is the “attentional drift” [22] at critical moments from the interaction with the horse in specific settings that poses dangers. The awareness or focus of an individual can drift and, when this happens, more automatic responses can emerge with less awareness for recalibrating risk [21,22]. Three examples are shown in Table 2.

**Table 2:** Examples from track-work riders and jockeys, equine veterinarians and equestrian competitors that illustrate the drifting awareness that increase the risk of injuries.

Track-Work Riders and Jockeys	Equine Veterinarians	Equestrian Competitors
Examples of how the awareness or focus of an individual can drift and, when this happens, more automatic responses can emerge with less awareness for recalibrating risk		
A track rider exercising a horse could be distracted by a discussion with a co-worker riding alongside and not notice a change at the racecourse, such as some new machinery. The horse could spook.	A vet could be distracted while performing an examination and explaining something to an owner. Meanwhile, the horse stands on the vet’s foot or kicks out.	A riding competitor is distracted by another horse’s behaviour and loses focus on his own horse’s reaction. Thus, the rider did not notice the fear building in his horse and not respond early enough to defuse it.

Research shows that inattention has been associated with an increased risk of crashing vehicles, such as cars or trucks [29,30,31], as well as with risky health behaviours, such as smoking tobacco [22]. Smokers routinely fail to accurately consider that smoking elevates their risk of cancer and cardiovascular diseases [22]. Therefore, risks for injury, death or illness increase when dissociation happens in contexts that are inappropriate and problematic. Similarly, it is feasible that dissociation may be a process that hinders human–horse risk.

Indeed, Hausberger *et al.* [26] reported that observational skills and attentional skills were pivotal in preventing accidents specific to humans and horses. Perhaps contrary to expectation, Hausberger *et al.*’s review [26] concluded that documented accidents did not decrease with the degree of human competence or accumulated experience with horses. Hitchens *et al.* [2] reported that jockeys over 35 years of age had increased rates of falling off horses if they had ridden earlier at the race meeting, suggestive perhaps of fatigue or attentional issues. However, prior evidence reviewed [2,4] indicates other factors such as being less experienced, having less accomplished horses and not wearing a helmet were associated with elevated risk, thus highlighting human experience and horse variables as risks for injuries. There is evidence of a clear decrease in fall rates over time, *i.e.*, with accumulated experience [32].

Thompson and Haigh [5] reported that horse riders “rarely described their own horses as dangerous or unpredictable” because of their experience and familiarity with these animals. However, fractious horse behaviour with a rapid flight response [18] can make horses dangerous in their responses around people [13,27]. Riders often fail to appreciate a change in the direction or the quality of their focus as critical for risk mitigation. Instead, they rely on experience as the predictor, which indeed could be a serious or even fatal error for risk appraisal. The specific context and loss of focus already pose two major risks, but a further issue, such as cognitive style, can also thwart accurate risk reappraisal.

## 4. A Global Cognitive Style Can Bring Specific Risks

A global cognitive style is a broad style of thinking that focuses on the bigger picture and is over-inclusive [20,21]. It lacks the specificity and filtering processes necessary for some thoughts or perceptions to be accurate, true, integrated and representative [20,21,33]. Again, this is a neutral term that relies on context for its utility. To illustrate this, a global overarching principle for life, say, regarding animal welfare (e.g., “animal abuse is wrong”), may indeed be helpful in protecting animals from harm. A metaphor for a global cognitive style could be seeing the forest but not the trees [20]. A sweeping broad view, such as a global cognitive style cannot be relevant for all circumstances, as it overlooks specific risks. A global cognitive style regarding accident risk could be problematic. In this vein, a study by Thompson and Haigh [9], when they investigated the use or lack of use of helmets, highlighted the global cognition of riders that “accidents happen”, “I can control risk” and “it does not feel right”. It is highly improbable that each of these statements could be accurate in all situations across horse care, preparation, handling practices, riding, track work, racing, competition venues, veterinary practices or the home environment. So, specific and focused adjustments for contexts and circumstances offer a critical opportunity to reappraise the process of risk assessment and management.

Without the context, focal point and specifics, the opportunity for cognitive errors in assessing risk increases [22]. Specificity is an antidote for a global cognitive error. As specific realistic risks are raised under focused states of awareness, decision-making can be altered to minimise or avoid the risk. When the cognitive error is acknowledged in a focused state, some people recalibrate their behaviours and choices. In his clinical work with people who engage in risky behaviours, Yapko [20,21,22] has documented the teaching of skills and routine recalibration of risks under focused states of awareness.

**Table 3:** Examples from track-work riders and jockeys, equine veterinarians and equestrian competitors that illustrate global cognitions that increase the risk of injuries.

Track-Work Riders and Jockeys	Equine Veterinarians	Equestrian Competitors
Examples of human global cognitions that elevate risk		
Global: Everyone runs risks in the workplace. It’s just bad luck if you get injured.	Global: Older mares don’t need to be scanned in crushes.	Global: Riding horses is no more dangerous than any other sporting activity.
Specific: Some risks in the racing industry can be identified, managed and avoided when safety protocols are followed.	Specific: Even when scanning an experienced broodmare, it would be sensible to reduce the risk of injury by using a crush, especially given it’s a veterinary examination that occurs less frequently and calls for extra handling skills.	Specific: Given that a horse’s response to fear is to flee, riding horses can elevate the risk of injury and mortality, especially when high speeds and jumping are involved.

A global cognitive style can ultimately be detrimental for the process of differentiation [20,21,22]. Differentiating and separating certain elements of experiences should be considered a core skill required for mitigating risk. This is especially so, given the cognitive differences between humans and horses, although any discussion of these differences, either real or putative, can be contentious. Humans have the ability to generalise learning across situations; in contrast, equitation scientists generally maintain that horses are context-specific in their approach, do not generalise immediately, and do not possess higher cognitive abilities [34,35,36,37]. A rider can train a horse to go forward in a certain place, but a horse, particularly a young one, associates all the cues (e.g., visual) specific to that particular place with the go-forward cue. Therefore, the younger horse may fail to go forward in another situation [35,36]. An example would be when the horse learns to travel through a water obstacle at home, but fails to generalise it to all water obstacles at different venues until all the other concomitant visual details are less relevant than the water itself [35]. It is critical that people who work with horses understand the intricate learning processes of horses and humans, especially given that horses can be context-specific [35,36] with a new skill and the rider may be global [20,21,22] in his approach. A context-specific horse with a global rider could quickly succumb to an elevated risk of injury. Examples are shown in Table 3.

People who work with horses and who have a global cognitive style, a loss of focus and who fail to consider the context, collectively or separately can have an elevated risk of injury or mortality. As the case builds across this article, the final problematic process is the ability to see risk only through one lens or focal point.

## 5. Using the Self as a Frame of Reference Hinders Flexibility in Risk Appraisal

The idea that one person’s perspective is fact can potentially contribute to the risk of injury. It is important to appreciate that each horse can have a repertoire of current behaviours that vary from the most recently observed [27]. More specifically, a given horse may adopt a certain response in one episode and a different response in the same environment because horses trial behaviours to cope with circumstances and do not always generalise [27,34,35,36]. If workers in the racing industry, veterinary practice or equestrian disciplines assume that horses will react in the same way as they have done previously, they are embracing a dangerously rigid approach to risk assessment and management.

Yapko [21] defined “using the self as a frame of reference” as perceiving or interpreting information from the person’s social learning history without consideration of alternative views. It can indicate a pattern of selective perception. So, being wedded to a particular view that is safety -focused may have an advantage but, plainly, adhering to a view that elevates risk (e.g., “I’ve ridden without a helmet before and I’ve been fine”) could be problematic [21]. A rigid style discounts the probability of the horse trialing a new behaviour, so riders may be surprised by the behaviour and delayed in their response. A flexible style (using other points of reference) would be more accommodating, allowing riders to respond quickly if they have the skills to cope with such behaviour and correct it. Examples are shown in Table 4.

**Table 4:** Examples from track-work riders and jockeys, equine veterinarians and equestrian competitors that illustrate self-referencing as a cognitive error that increases the risk of injuries.

Track-Work Riders and Jockeys	Equine Veterinarians	Equestrian Competitors
Examples for the idea of self-referencing as an assessment point		
A horse at home has no history of rearing. On race day, it is exposed to the public address system and rears. The stable attendant is surprised, dragged sideways and delays making a response.	A horse attends the vet’s premises for treatment. The owner says the horse has never kicked out before, but in an unfamiliar environment while undergoing veterinary procedures, such as injections, it kicks out as it is insecure and unwell.	A horse spooks at a yellow garbage bin in the warm-up arena at a new competition venue. The horse does not typically shy at home. The rider is perplexed and caught unawares. The rider’s self-referencing ideation can heighten the risk of a fall or provide delays in signaling the horse to move forward (e.g., faster with leg speed or longer in the stride) and maintain focus.

## 6. Conclusions and Future Direction

Drawing on the available literature, our analysis has found that while the four critical concepts for human–horse risk mitigation; namely contextual relevance, inattention or loss of focus, global cognitions and referencing on the basis of past history, are not problematic *per se*, it is the context in which they are applied that is paramount when assessing risk for human–horse interactions. We have provided examples to demonstrate how each of these risk factors relate to the three high at-risk groups identified earlier in this paper; track-work riders and jockeys, equine veterinarians and equestrian competitors.

We posit that to reduce risk, the formation of new associations that realistically appraise the risk in an ongoing manner and prompt actions that are context-specific, focused, and flexible offer a sensible approach [20,21,22]. In the pragmatic sense, using metaphors and hypnosis to alter the cognitive, emotional and behavioural options of paying attention to horses’ cues and behaviours in specific scenarios (e.g., transport loading, racetrack behaviour, or injection as part of veterinary practice) could be used. Establishing a tool-kit with information on how horses learn and respond (e.g., habituation, learning theory; pressure and release, positive reinforcement) could be of use, albeit to illustrate key points, the language could be modified for lay people. Metaphors and hypnosis could emphasise and promote the advantages of acquiring the specific skill-set or to make adjustments in the skill(s) used (e.g., appropriate use of negative reinforcement, such as via the bit as a stop cue; wither scratching for an anxious horse; allowing the horse to assess an aversive object, subject to the arousal state of the horse [38]). Baseline and post-intervention measures could be developed to test for statistical or clinically relevant change (*i.e.*, of practical or applied value in everyday life) [39].

The awareness of risk factors, the association of these risk factors with workers in different parts of the equine industry who are most at-risk, and identifying the implications if these risks translate into actual incidents, are all important steps in reducing equine-related accidents. However, it is important to go beyond these steps and suggest that preliminary solutions that may be developed in the future into a more comprehensive model built on the foundations outlined in this paper. For example, dissociation in a new environment, or even in a familiar environment, can pose risks due to the inattention that arises in the human–horse interaction, specifically distraction from monitoring the horse’s responses. Subsequently, the human response will inevitably be delayed. Focused awareness is central to this risk-mitigation model. The human expectation that a horse will respond similarly in all scenarios without the human appropriately assimilating the requirements of the horse or specific situations could elevate the risk of human injury or death. Choosing a poorly matched strategy for the situation could also be disastrous. Combining the contextual, cognitive errors with a rigid self-referenced point of view and a lack of focused attention helps to clarify how risk rapidly emerges, and how others may repeatedly become at risk over time.

Essentially, many workers in the racing industry and people across all equestrian disciplines may be unaware of some of the critical requirements for realistically assessing the risk of injury. This paper offers crucial suggestions on what is important and how to form a pattern of interruption [22] to help to address the risks that equine industry workers and humans in equestrian disciplines currently face.
